# Magnetic Resonance-Guided Radiotherapy for Unresectable Hepatocellular Carcinoma with Bile Duct Tumor Thrombus: A Case Series and Review of Treatment Options

**DOI:** 10.3390/curroncol33070425

**Published:** 2026-07-16

**Authors:** Nam Kyu Kang, So Jung Lee, Hye Jin Kang, Hun-Joo Shin, Jung Hyun Kwon, Soon Kyu Lee, Myungsoo Kim

**Affiliations:** 1 Department of Radiation Oncology, Incheon St. Mary’s Hospital, College of Medicine, The Catholic University of Korea, Seoul 06591, Republic of Korea; kng1032@catholic.ac.kr (N.K.K.); sj_lee@catholic.ac.kr (S.J.L.); kanghj@catholic.ac.kr (H.J.K.); 10611561@cmcnu.or.kr (H.-J.S.); 2 Department of Internal Medicine, Incheon St. Mary’s Hospital, College of Medicine, The Catholic University of Korea, Seoul 06591, Republic of Korea; doctorkwon@catholic.ac.kr (J.H.K.); blackiqq@catholic.ac.kr (S.K.L.); 3 The Catholic University Liver Research Center, College of Medicine, The Catholic University of Korea, Seoul 06591, Republic of Korea

**Keywords:** hepatocellular carcinoma, bile duct tumor thrombus, MR-guided radiotherapy, stereotactic body radiotherapy, biliary obstruction

## Abstract

Bile duct tumor thrombus (BDTT) is a rare manifestation of hepatocellular carcinoma (HCC) that causes obstructive jaundice and is associated with a poor prognosis, although a treatment algorithm has yet to be established. We review the treatment landscape for HCC with BDTT, including surgical, transarterial, systemic, and radiotherapy options, and report, to our knowledge, the first case series of magnetic resonance-guided radiotherapy (MRgRT) for this condition. Four patients with unresectable HCC and BDTT were treated on a 0.35-T MR-linac with real-time cine-MRI gating (50 Gy in 5 fractions, *n* = 3; 40 Gy in 5 fractions, *n* = 1), with tumor response assessed by modified RECIST. All patients completed treatment without interruption. The best response was complete response in two patients and partial response in two, and obstructive jaundice resolved in both affected patients. The maximum radiation-attributed toxicity was a transient grade 3 bilirubin elevation that resolved without biliary intervention, and no treatment-related deaths occurred. Overall survival ranged from 5.5 to 29 months, with the two Child-Pugh class A patients without portal vein tumor thrombosis surviving 22 and 29 months. MRgRT for HCC with BDTT appears feasible with acceptable short-term safety and merits prospective evaluation.

## Simple Summary

Liver cancer can sometimes grow into the bile ducts, forming a bile duct tumor thrombus. This rare complication causes jaundice and limits treatment options, as fewer than one in ten patients qualify for surgery. Radiation therapy has been explored using proton beams, but MR-guided radiotherapy, which allows doctors to see the tumor in real time during treatment, has never been reported for this condition. We treated four patients using an MR-guided radiation system that tracks tumor movement caused by breathing and automatically pauses the beam when the tumor moves out of the target zone. All four patients completed treatment successfully. Two achieved complete disappearance of the tumor thrombus and two showed partial shrinkage. Jaundice resolved in both patients who had it before treatment. No deaths were related to the radiation treatment. These preliminary results suggest that MR-guided radiotherapy is a feasible and well-tolerated option that deserves further study.

## 1. Introduction

Hepatocellular carcinoma (HCC) is the third leading cause of cancer-related mortality worldwide, accounting for approximately 758,000 deaths annually [1]. Although the majority of patients present with parenchymal hepatic disease, a clinically distinct subset develops tumor invasion into the intrahepatic or extrahepatic bile duct system, resulting in bile duct tumor thrombus (BDTT). This entity, also designated icteric-type HCC, carries a disproportionately poor prognosis relative to HCC without bile duct involvement, with a median overall survival (OS) of 1.6 to 4.3 months under best supportive care [2]. The pathological hallmark is progressive intraductal tumor extension with consequent biliary obstruction, manifesting as obstructive jaundice, hemobilia, and recurrent cholangitis [2,3].

Evidence from published series has demonstrated that the incidence of BDTT varies widely, ranging from 0.4% to 12.9%, with this variation likely attributable to differences in diagnostic criteria, imaging modalities, and case ascertainment methods [2,4]. Establishing the diagnosis requires careful differentiation from perihilar cholangiocarcinoma, which presents with an identical clinical syndrome. Elevated serum alpha-fetoprotein (AFP) levels, underlying viral hepatitis, and characteristic arterial-phase enhancement on dynamic imaging favor the diagnosis of HCC with BDTT [4]. The Ueda classification system categorizes BDTT into types I-IV according to the proximal level of biliary involvement [5].

The optimal management of HCC with BDTT remains poorly defined, and no consensus treatment algorithm has been established. Surgical resection with bile duct thrombectomy represents the only potentially curative option; however, fewer than 11% of patients are eligible for this approach at presentation [4,6]. Transarterial chemoembolization (TACE) and systemic therapies, including atezolizumab plus bevacizumab (Atezo/Bev) and lenvatinib, constitute the principal non-surgical options; however, both are substantially limited in this population. TACE carries a risk of sloughing of necrotized thrombus and subsequent biliary complications, while contemporary immunotherapy-based regimens are frequently precluded by hyperbilirubinemia secondary to biliary obstruction [7,8,9]. Radiotherapy has been explored as an adjunctive or primary modality for locally advanced HCC with macrovascular invasion, although published evidence specifically addressing BDTT remains limited to a small number of case reports and two proton beam therapy (PBT) series, and no experience with magnetic resonance-guided radiotherapy (MRgRT) has been reported to date [10,11].

MRgRT has emerged as a distinct image-guidance approach for delivering stereotactic body radiotherapy (SBRT) to hepatic tumors, enabling direct real-time target visualization through on-board magnetic resonance imaging (MRI) during beam delivery and real-time cine-MRI gating throughout each treatment session [12]. These capabilities are directly relevant to the central hepatobiliary location characteristic of BDTT, where precise targeting adjacent to the bile duct confluence, portal vein, and gastroduodenal structures is critical to minimize the risk of treatment-related biliary toxicity [13]. Our institution has previously demonstrated the feasibility and efficacy of MRgRT for HCC with portal vein tumor thrombosis (PVTT) [14], a closely related entity sharing anatomical proximity to central hepatic structures. To our knowledge, we report the first case series of MRgRT specifically targeting HCC with BDTT, derived from a prospectively maintained institutional database. We additionally review the treatment landscape for this rare entity, with particular emphasis on the evolving role of radiotherapy.

## 2. Methods

### 2.1. Search Strategy

We conducted a narrative review of the published literature on the treatment of HCC with BDTT. PubMed and Embase were searched on 11 May 2026, using terms related to HCC with bile duct involvement in combination with terms for individual treatment modalities, including surgery, transarterial therapy, systemic therapy, and radiotherapy. The search was restricted to English-language articles without date limitations. Titles and abstracts were screened for relevance, and full texts of potentially eligible articles were reviewed. Additional publications were identified through manual review of the reference lists of retrieved articles. Studies were selected based on their relevance to the epidemiology, pathophysiology, diagnosis, or treatment of HCC with bile duct involvement. Given the limited published experience, all identified studies addressing radiotherapy for this entity were included regardless of study design or sample size.

### 2.2. Case Series

This retrospective case series was approved by the Institutional Review Board of Incheon St. Mary’s Hospital, The Catholic University of Korea (IRB No. C26RISI0090), and the requirement for individual informed consent was waived owing to the retrospective study design. All patients with unresectable HCC and histologically or radiologically confirmed BDTT who underwent MRgRT at our institution between January 2019 and December 2025 were identified from a prospectively maintained radiation oncology registry. Histological confirmation of HCC was obtained in two patients (Patients 1 and 4) through endoscopic retrograde cholangiopancreatography-guided bile duct biopsy. Immunohistochemistry was positive for AFP and hepatocyte antigen in Patient 1 and for AFP and glypican-3 in Patient 4. In both cases, the pathological diagnosis was reported as atypical cells consistent with carcinoma. In the remaining two patients, the diagnosis was based on imaging findings. All four cases were reviewed and managed by a multidisciplinary team. Radiological diagnosis required the presence of an intraductal soft-tissue lesion contiguous with the parenchymal HCC, demonstrating arterial-phase enhancement on contrast-enhanced MRI or computed tomography (CT), together with elevated serum AFP levels and underlying chronic liver disease, in the absence of imaging features suggestive of perihilar cholangiocarcinoma. Patients in whom BDTT was an incidental finding and PVTT was the sole treatment target were excluded. Four patients met the inclusion criteria and were included in this analysis. Biliary drainage was performed prior to MRgRT in patients presenting with active cholangitis or hyperbilirubinemia, at the discretion of the treating multidisciplinary team.

#### 2.2.1. MRgRT Technique

All four patients were treated on a 0.35-T MR-guided linear accelerator (ViewRay MRIdian; ViewRay, Inc., Oakwood Village, OH, USA), following the institutional hepatic MRgRT workflow reported previously [14]. At simulation, each patient lay supine with the arms raised overhead and was secured in a Vac-Lok immobilization device (CIVCO, Coralville, Iowa, USA). Magnetic resonance (MR) images were captured using a balanced steady-state free precession (bSSFP) sequence (TrueFISP) without gadolinium contrast, at a 3 mm slice thickness, each acquisition spanning a 25 s breath-hold at end-exhalation. Once the treating radiation oncologist judged the image quality adequate, a short 10 s cine sequence in the sagittal plane was obtained to confirm that the target could be tracked in real time. A contrast-enhanced CT scan was subsequently acquired within 30 min in the same position and was coregistered with the MR dataset to provide electron-density information. Using the MR simulation images, with reference to both the CT and pre-treatment gadoxetic acid (Primovist)-enhanced diagnostic MRI co-registered in MIM (MIM Software Inc., Cleveland, OH, USA), a radiation oncologist delineated the gross tumor volume (GTV) to encompass the intraductal thrombus and any directly contiguous hepatic parenchymal mass. No clinical target volume expansion was applied, and the planning target volume (PTV) was instead formed by enlarging the GTV with an isotropic 5 mm margin. The same 5 mm isotropic margin was applied to both the intraductal thrombus and any contiguous parenchymal mass. This margin accounted for residual setup and target delineation uncertainty, with respiratory motion managed by real-time cine-MRI gating rather than by an internal target volume margin. Online adaptive replanning, available on the MRIdian platform, was not performed in any fraction, as setup imaging confirmed adequate target coverage at each fraction. Throughout delivery, sagittal cine-MRI provided real-time gating, automatically halting the beam whenever target excursion exceeded 3% of the PTV envelope. The sagittal gating plane was selected at each fraction to encompass the region of the target demonstrating the greatest respiratory excursion. In patients whose GTV encompassed both the parenchymal mass and the contiguous thrombus, the gating plane was positioned through the center of the combined target, which moved as a single unit during respiration. For Patient 2, whose target consisted of the intraductal thrombus alone, the gating plane was centered on the thrombus. Three patients received 50 Gy and one received 40 Gy, each delivered in five fractions on consecutive weekdays, corresponding to biologically effective doses (BED_10_) of 100 Gy and 72 Gy, respectively. The radiation beam was delivered using a step-and-shoot IMRT technique. Across all 20 treatment fractions, the median total treatment time per fraction was 23.2 min (range, 20.5–36.4). Median beam-on time was 5.44 min (range, 5.01–5.59), and the median gating duty cycle was 87.3% (range, 83.0–89.9%), indicating efficient beam delivery throughout the gating period. Intra-fraction beam holds, triggered by transient target excursion beyond the 3% gating boundary during respiratory motion, occurred in 6 of 20 fractions (30%). All were promptly resumed and completed within the same session without rescheduling. No fraction was permanently terminated due to technical failure. Although the intraductal thrombus itself was not always distinctly visualized on cine-MRI, the combined target contour remained sufficiently identifiable to permit reliable target tracking throughout treatment.

#### 2.2.2. Outcome Assessment

Tumor response was evaluated according to the modified Response Evaluation Criteria in Solid Tumors (mRECIST) for HCC [15] on follow-up contrast-enhanced CT or MRI performed at approximately one month after treatment completion and at 2–3-month intervals thereafter. Toxicity was graded according to the Common Terminology Criteria for Adverse Events version 5.0 (CTCAE v5.0). Consistent with the attribution approach applied in our institutional series of MRgRT for PVTT [14], radiation-attributed hepatobiliary toxicity was distinguished from disease-related hepatic dysfunction by the treating radiation oncologist together with a hepatologist based on three criteria: the temporal relationship between bilirubin change and radiotherapy; the presence or absence of concurrent radiographic tumor progression or new biliary obstruction on cross-sectional imaging; and the reversibility of the abnormality. Bilirubin elevations that coincided with radiographically confirmed tumor progression or progressive biliary obstruction were classified as disease-related rather than radiation-attributed. The maximum grade of toxicity attributable to radiotherapy is reported separately from the maximum observed bilirubin value for each patient. OS was defined as the interval from the first day of MRgRT to death from any cause or last follow-up. Data were censored on 31 December 2025. Given the descriptive nature of this case series, no formal statistical analysis was performed. Outcomes are reported as individual patient data.

## 3. Literature Review

### 3.1. Epidemiology and Pathophysiology of BDTT

**Figure 1:**
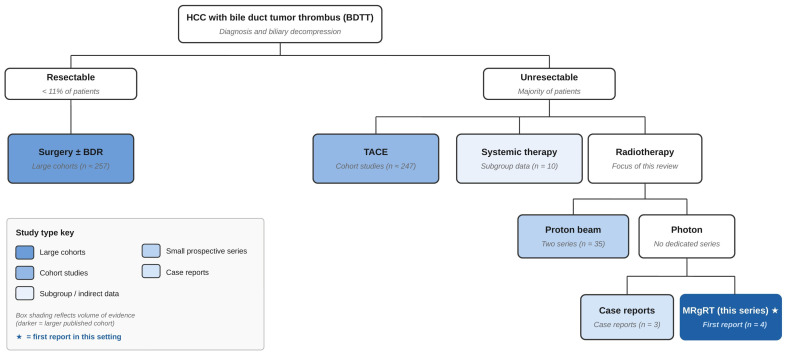
Treatment landscape for hepatocellular carcinoma with bile duct tumor thrombus. Treatment options are stratified by resectability: fewer than 11% of patients are surgical candidates at presentation, and the majority require non-surgical approaches. Within the radiotherapy branch, evidence is stratified by beam modality, with no dedicated series reported outside proton beam therapy prior to the present study. Box shading reflects the volume of published evidence in the BDTT-specific context (darker = larger cohorts); subtitles denote study type and sample size. The star (★) denotes the first report in this setting. BDR, bile duct resection; BDTT, bile duct tumor thrombus; MRgRT, magnetic resonance-guided radiotherapy; TACE, transarterial chemoembolization.

The overall treatment landscape for HCC with BDTT is illustrated schematically in Figure 1. According to a previous review [4], HCC with BDTT is an uncommon clinical entity, first described by Mallory et al. in 1947 and subsequently designated icteric-type hepatocarcinoma by Lin et al. in 1975, owing to the characteristic presenting symptom of obstructive jaundice. Its reported incidence ranges between 0.4% and 12.9% in surgical and autopsy series, with the wide variation reflecting differences in diagnostic criteria, imaging modalities, and case ascertainment across institutions [2,4]. Jaundice is the most common presenting symptom, occurring in 53% of patients across published series, and serum AFP levels exceed 400 ng/mL in approximately 58% of cases [4]. Males are more frequently affected, accounting for approximately 83% of reported patients [4]. Beyond its low incidence, HCC with BDTT is characterized by a more aggressive biological profile. Compared with patients with HCC without BDTT, those with BDTT exhibit significantly higher rates of poorly differentiated histology (odds ratio [OR] 1.88; 95% confidence interval [CI] 1.15–3.05), lymphovascular invasion (OR 4.85; 95% CI 2.73–8.61), and portal vein invasion (OR 5.31; 95% CI 3.87–7.28) in a pooled analysis of histopathological studies [7]. Concurrent PVTT is present in approximately 67% of cases, likely reflecting the anatomical co-enclosure of the portal vein and bile duct within the Glisson sheath [2].

The pathogenic mechanism of BDTT remains poorly understood. The most widely accepted hypothesis proposes that tumor cells directly invade the intrahepatic bile duct from the adjacent parenchymal mass, with subsequent proximal extension of the tumor thrombus toward the common bile duct [3]. Alternative pathways include tumor dissemination via peribiliary microvessels, lymphatics, or arteriovenous shunts [3]. Once the thrombus extends to first-order or more proximal bile ducts, biliary obstruction ensues, manifesting as obstructive jaundice, hemobilia, and recurrent cholangitis [2]. Establishing the diagnosis requires differentiation from perihilar cholangiocarcinoma, which presents with a similar clinical syndrome. On dynamic contrast-enhanced imaging, BDTT typically demonstrates arterial-phase hyperenhancement with portal venous washout, mirroring the enhancement characteristics of the parenchymal HCC, whereas cholangiocarcinoma more commonly shows progressive periductal enhancement and ductal wall thickening without a discrete expansile intraductal mass [2,16]. Elevated serum AFP levels, the presence of underlying viral hepatitis, and the coexistence of a contiguous parenchymal mass favor the diagnosis of BDTT. Histopathological confirmation remains necessary in diagnostically ambiguous cases [3]. Further complicating diagnosis, preoperative detection of BDTT remains challenging even in the presence of characteristic imaging features. In a multicenter study of 30 patients with B1–B3 BDTT, nearly half were misdiagnosed before surgery, and intraductal thrombus was not identified on preoperative imaging in any patient with type B1 disease; however, intrahepatic bile duct dilatation served as an indirect marker, with a sensitivity of 93% and specificity of 99% for the diagnosis of BDTT [16]. The Ueda classification system is most commonly used to classify BDTT, stratifying intraductal extension into types I-IV according to the proximal extent of biliary involvement [5]. A simpler dichotomous classification distinguishing central type (involving the common hepatic duct or first-order bile duct) from peripheral-type disease (confined to second-order or more distal branches) has also been used, including in recent radiotherapy series, and has been associated with OS in surgical cohorts, with central type carrying a poorer prognosis [6].

### 3.2. Surgical Treatment

Surgical resection with concurrent bile duct thrombectomy or bile duct resection (BDR) represents the only potentially curative option for HCC with BDTT. In a Korean multicenter series of 73 patients who underwent resection with curative intent, the one-, three-, five-, and ten-year OS rates were 76.5%, 41.4%, 32.0%, and 17.0%, respectively. R0 resection was achieved in 78.1% of cases [17]. Preoperative biliary decompression was required in 45.2% of patients [17]. Nevertheless, fewer than 11% of patients present with resectable disease, owing to the frequent coexistence of advanced hepatic dysfunction, multifocal disease, and extrahepatic spread at the time of diagnosis [4,6].

The role of concurrent extrahepatic BDR at the time of hepatectomy remains controversial. A meta-analysis of 12 studies including 355 patients demonstrated comparable one-, three-, and five-year OS rates between the BDR and non-resection groups. However, BDR was associated with significantly improved postoperative recurrence-free survival (RFS) at all time points (one-year RFS: OR 0.38, 95% CI 0.22–0.65; three-year RFS: OR 0.40, 95% CI 0.22–0.72) [3]. A propensity score-matched analysis of a multicenter cohort of 1585 patients (227 with BDTT) confirmed that BDTT was an independent risk factor for reduced RFS after hepatectomy [18]. Based on these data, current practice favors an individualized surgical approach: when BDTT is confined within the hepatic resection plane, en bloc removal with the primary tumor is sufficient; when the thrombus extends to the extrahepatic bile duct but can be separated from the ductal wall, thrombectomy through choledochotomy is preferred; and when the thrombus adheres tightly to the bile duct wall, concurrent BDR is recommended to minimize the risk of local recurrence [3]. The largest published surgical series to date included 257 patients from 32 centers in Korea and Japan and reported five-year OS and recurrence rates of 43.6% and 74.2%, respectively [19]. In this cohort, concurrent BDR was associated with improved OS compared with thrombectomy alone (hazard ratio 0.61; 95% CI 0.38–0.99; *p* = 0.044), and R0 resection was the strongest determinant of long-term outcome [19]. An alternative surgical strategy, in which the thrombus is approached and extracted prior to resection of the parenchymal mass, has been reported to yield OS comparable to that achieved with BDR (42.7 vs. 33.6 months; *p* = 0.653) while avoiding the morbidity associated with extrahepatic duct reconstruction, suggesting that the sequence of surgical steps may influence biliary contamination and local recurrence [20]. A multicenter study of 138 patients who underwent hepatectomy with preservation of the extrahepatic bile duct reported a median OS of 28.6 months and RFS of 8.9 months. R0 resection and major vascular invasion were identified as independent prognostic factors [21]. A multicenter analysis of 1021 patients who underwent R0 resection identified bile duct invasion as an independent predictor of OS and proposed reclassifying HCC with BDTT without macrovascular invasion as Barcelona Clinic Liver Cancer (BCLC) stage B and HCC with BDTT and macrovascular invasion as BCLC stage C, reflecting the prognostic relevance of biliary involvement beyond current staging conventions [22].

High postoperative recurrence rates of 43–50% within the first year highlight the need for adjuvant locoregional strategies following resection [4].

### 3.3. Transarterial and Locoregional Therapies

In patients ineligible for surgical resection, TACE and hepatic arterial infusion chemotherapy (HAIC) have been explored as palliative options following adequate biliary decompression. In the largest reported cohort of 247 consecutive patients with BDTT, repeated TACE was identified as the most frequently used non-surgical treatment (42.5%), achieving a median OS of 6.0 months in patients with BCLC stage C [2]. Successful biliary drainage before HCC-directed treatment was independently associated with improved OS, with median survival of 12.9 months versus 1.5 months among patients without successful drainage [2].

A serum total bilirubin concentration ≥3 mg/dL is recognized as an important predictor of prolonged hospitalization after TACE [7,23]. Because BDTT may undergo complete necrosis following superselective TACE, sloughing of the necrotic thrombus into the common bile duct is a well-established complication requiring endoscopic removal. In addition, intraductal hemorrhage during TACE is a known complication that may necessitate biliary drainage or bland embolization to achieve hemostasis [7]. TACE was associated with a median OS of 12.2 months in patients managed according to contemporary protocols [23]. HAIC using an indwelling catheter-port system with cisplatin-based or oxaliplatin-based regimens has been employed in patients with HCC beyond conventional TACE indications, including those with concurrent portal vein and bile duct invasion. A single case report described a complete resolution of the intraductal thrombus following intra-arterial cisplatin infusion; however, systematic evidence supporting this approach in patients with BDTT remains lacking [7]. Transarterial radioembolization with yttrium-90 microspheres has demonstrated activity in HCC with major vascular invasion, with median OS ranging from 9 to 17 months depending on the extent of portal vein involvement. However, dedicated data evaluating its efficacy in patients with BDTT have yet to be reported [7].

### 3.4. Systemic Therapy

First-line systemic therapy with Atezo/Bev or lenvatinib is now the established standard of care for unresectable advanced HCC [8,9]. However, patients with BDTT were not specifically represented in the pivotal studies establishing these regimens. The IMbrave150 trial restricted enrollment to patients with Child-Pugh class A liver function [8], whereas the REFLECT trial explicitly excluded patients with obvious bile duct invasion [9]. Hyperbilirubinemia, a common consequence of BDTT, also frequently precludes safe initiation of either regimen in clinical practice. Accordingly, direct evidence supporting contemporary immunotherapy-based regimens in this population is lacking, and their safety and efficacy in the context of active biliary obstruction remain undefined. In clinical practice, systemic therapy is therefore typically deferred until biliary obstruction has been adequately relieved by endoscopic or percutaneous drainage, or until locoregional treatment has achieved sufficient tumor regression to permit normalization of serum bilirubin levels.

To date, the only published study specifically evaluating outcomes of systemic therapy in HCC with bile duct invasion was a retrospective cohort analysis of 175 patients treated with sorafenib, of whom 10 had bile duct invasion [24]. Among the bile duct invasion subgroup, the median OS was 14.1 months, and the disease control rate was 70.0%. Five of the ten patients developed cholangitis during treatment, requiring endoscopic biliary intervention, and two required biliary drainage before sorafenib initiation to enable safe treatment delivery [24]. The authors reported that proactive endoscopic management of biliary complications during sorafenib treatment was associated with shorter treatment interruption periods, highlighting that maintenance of biliary patency represents an ongoing therapeutic requirement rather than a one-time prerequisite [24]. These observations underscore both the potential activity of systemic agents in this population and the critical importance of integrated multidisciplinary biliary management before and throughout treatment. Similarly, the HIMALAYA trial of tremelimumab plus durvalumab did not report outcomes specific to bile duct involvement and excluded patients with main PVTT, leaving the efficacy of this regimen in BDTT undefined [25]. A single published case report described a 68-year-old man with advanced HCC and BDTT extending to the hepatic duct bifurcation who achieved a complete response (CR) according to RECIST criteria six months after initiating tremelimumab plus durvalumab. Treatment was subsequently discontinued owing to immune-related type 1 diabetes mellitus and diabetic ketoacidosis [26]. To date, this represents the only published report of CR to immune checkpoint inhibitor (ICI)-based therapy in HCC with BDTT, although no generalizable conclusions can be drawn from a single case.

A complementary rationale for the use of radiotherapy in this setting arises from its potential synergy with systemic immune-based therapy. Radiotherapy induces immunogenic tumor cell death, leading to the release of tumor-associated antigens and damage-associated molecular patterns that promote dendritic cell activation and T-cell priming. In addition, it can shift the immunosuppressive hepatic tumor microenvironment toward a state more responsive to ICI-mediated blockade [27]. In preclinical models, hypofractionated stereotactic regimens appear to be more immunogenic than conventional fractionation. Isolated reports have documented out-of-field (abscopal) responses when radiotherapy is combined with checkpoint inhibition in HCC [27,28]. A recent systematic review and meta-analysis of external radiotherapy combined with ICIs in advanced or unresectable HCC, encompassing 16 studies and 633 patients, reported a pooled objective response rate (ORR) of 54.4% (95% CI 46.8–62.0%), compared with ORRs of 15–30% reported for ICI monotherapy in advanced HCC [29].

These findings are directly relevant to HCC with BDTT. Because hyperbilirubinemia resulting from biliary obstruction frequently precludes the safe initiation of ICI-based regimens, locoregional radiotherapy may serve a dual function in this population by achieving local tumor control and restoring biliary patency while simultaneously priming an antitumor immune response that may be consolidated with sequential systemic therapy once serum bilirubin levels have normalized. The optimal sequencing and dose-fractionation of radiotherapy and checkpoint inhibition in HCC remain undefined and have not been evaluated in any BDTT-specific cohort. The present series contributes preliminary observational data to this unresolved question.

### 3.5. Radiotherapy for HCC with BDTT

Radiotherapy for HCC with BDTT has not been systematically evaluated, with existing literature limited to a small number of case reports and two dedicated PBT series. A fundamental challenge in the radiotherapeutic management of BDTT is its anatomical location within the central hepatobiliary tract. Osmundson et al. demonstrated that the presence of an indwelling biliary stent during stereotactic body radiotherapy was among the strongest nondosimetric predictors of grade ≥ 3 hepatobiliary toxicity. Furthermore, dosimetric parameters, including the volume of the central hepatobiliary tract receiving a BED_10_ ≥ 72 Gy, were independently associated with biliary stricture and infection requiring intervention [13].

Prior to the dedicated PBT series described below, the published radiotherapy literature for this entity consisted of only two isolated case reports and one mixed cohort. Sung et al. reported a patient with nonresectable icteric-type HCC and common hepatic duct thrombus who was treated with external beam radiotherapy to 38 Gy in 10 fractions. Following treatment, serum bilirubin declined from 30.0 to 1.7 mg/dL with concomitant tumor reduction, and the patient subsequently received TACE and remained alive at 8 months [30]. Park et al. reported a case treated with CyberKnife SBRT to 37 Gy in 3 fractions, achieving partial regression of the primary tumor and normalization of serum bilirubin levels [31]. Kato et al. reported a heterogeneous cohort of patients with advanced HCC treated with CyberKnife SBRT, in which a subgroup with vascular or bile duct invasion achieved a 50% radiological response rate, although outcomes specific to BDTT were not separately reported [32]. In each of these reports, effective local tumor control was associated with restoration of biliary patency. To date, no published series has evaluated radiotherapy for BDTT using real-time MR guidance.

Lee et al. reported outcomes in 20 patients treated with PBT to a dose of 72.6 GyRBE in 22 fractions [10]. The one-year cumulative local recurrence rate was 5.3%, while the one- and two-year OS rates were 79.4% and 53.3%, respectively. Cholangitis was a major concern, with eight patients requiring antibiotic therapy and two dying from cholangitis-related sepsis. The one-year cholangitis-free survival was 55.0% [10]. Four patients developed radiation-induced liver disease, and eight experienced grade 2 or 3 gastroduodenal ulcers [10]. Iizumi et al. subsequently reported 15 patients treated with PBT using the same 72.6 GyRBE in 22 fractions [11]. The one-, two-, and three-year OS rates were 80.0%, 58.7%, and 40.2%, respectively, with a corresponding one-year local control rate of 93.3%. One patient developed grade 3 cholangitis, and no grade ≥ 4 toxicities were observed [11].

Collectively, these two series enrolled 35 patients and constitute the entirety of published radiotherapy-specific evidence for HCC with BDTT. All 35 patients were treated with PBT, a modality that is not universally available and is associated with substantially greater logistical and financial barriers than conventional photon radiotherapy. These two series adopted different classification frameworks (the Ueda system in Lee et al. [10] and the central/peripheral system in Iizumi et al. [11]) and were published independently without cross-citation, underscoring the fragmented and underdeveloped nature of the radiotherapy literature for this entity. No MR-guided radiotherapy series specifically addressing BDTT has been published to date. By enabling direct real-time visualization of intraductal thrombus and real-time cine-MRI gating during beam delivery, MR-guided radiotherapy may improve targeting precision in the central hepatobiliary location of BDTT and forms the basis for the present series. The current treatment landscape is illustrated schematically in Figure 1, and representative treatment outcomes across all modalities are summarized in Table 1.

**Table 1:** Representative Studies on Treatment Outcomes for Hepatocellular Carcinoma with Bile Duct Tumor Thrombus or Bile Duct Invasion.

Study (Year)	N	Median Tumor Size (cm)	BCLC C (%)	Key Outcomes	Resectability	Biliary Drainage Required	Limitation/Note
**Surgical Resection**							
Moon et al., 2013 [17] World J Surg	73	NR	NR	OS: 1y 76.5%, 3y 41.4%, 5y 32.0%, 10y 17.0%; R0: 78.1%	<11% eligible	45.2%	Korean MC; Ueda type 2–3 only; pre-ICI era
Kim et al., 2020 [19] Ann Surg	257	NR	NR	OS: 5y 43.6%; recurrence: 5y 74.2%; BDR vs thrombectomy: HR 0.61 (*p* = 0.044)	<11% eligible	NR	Largest surgical series; 32 centers (Korea–Japan); 1992–2014
Wu et al., 2022 [18] Ann Surg Oncol	227 (PSM)	NR	NR	5y OS: 39.0% (BDTT) vs. 38.7% (non-BDTT), *p* = 0.588; BDTT: independent predictor of worse RFS	Surgical cohort	NR	Prognostic study; no treatment comparison; *n* = 1585 total
**Transarterial and Locoregional Therapies**							
An et al., 2017 [2] Clin Mol Hepatol	247	8.0	66.8%	MST (all): 4.1 mo; BCLC C—surgery: 11.5 mo, TACE: 6.0 mo; Biliary drainage: 12.9 vs. 1.5 mo (*p* < 0.001)	10.9% received surgery	35.6%	Mixed BCLC stages; pre-ICI era; 67.2% concurrent PVTT
Choi et al., 2015 [23] Cardiovasc Intervent Radiol	53	NR	NR	TACE for BDTT: MST 12.2 mo; T-bili ≥ 3 mg/dL: predictor of prolonged hospitalization (HR 4.341, *p* = 0.022)	Unresectable (all TACE)	47% (25/53)	TACE-only; central BDI; single center
**Systemic Therapy**							
Tanaka et al., 2020 [24] Oncology	10 (BDI subgroup)	NR	BCLC B/C mixed	Sorafenib: MST 14.1 mo; DCR 70.0%; Cholangitis in 5/10 pts	Unresectable (all sorafenib)	2/10 pts	Smallest BDI subgroup (*n* = 10); sorafenib only; no ICI data
**Radiotherapy—Photon**							
Sung et al., 2001 [30] Chang Gung Med J	1	NR	NR	Serum bilirubin declined from 30.0 to 1.7 mg/dL; tumor reduction; patient alive at 8 months; 38 Gy in 10 fractions (EBRT)	Unresectable	NR	Case report; photon EBRT; non-stereotactic fractionation; pre-ICI era
Park et al., 2011 [31] Korean J Intern Med	1	NR	NR	Partial regression of primary tumor and BDTT; bilirubin normalization; 37 Gy in 3 fractions (CyberKnife SBRT); combined with TACE	Unresectable	NR	Case report; photon SBRT (CyberKnife); no long-term follow-up reported
**Radiotherapy—Proton Beam Therapy**							
Lee et al., 2022 [10] Cancers	20	6.3	65%	OS: 1y 79.4%, 2y 53.3%; MST 19.9 mo; LC: 1y 94.7%; Cholangitis requiring antibiotics: 8/20; 2 cholangitis-related deaths	Unresectable (all PBT)	35% (7/20)	Single PBT center (Taiwan); Ueda classification; high cholangitis rate
Iizumi et al., 2023 [11] BMC Gastroenterol	15	4.0	NR	OS: 1y 80.0%, 2y 58.7%, 3y 40.2%; LC: 1y 93.3%; No Gr4+ toxicity; Gr3 cholangitis: 1 pt (6.7%)	Unresectable (all PBT)	27% (4/15)	Single PBT center (Japan); central/peripheral classification; no cross-citation with Lee 2022 [10]

Table studies listed are representative reports chosen to illustrate outcomes across the principal therapeutic modalities and do not constitute a systematic or exhaustive compilation. Patient populations are not directly comparable across studies owing to differences in study design, patient selection criteria, BCLC stage distribution, tumor size, and treatment era. Survival outcomes should be interpreted with caution. The present MRgRT series (*n* = 4) is detailed separately in Table 2 and Table 3. Kato et al. [32] reported a mixed cohort of advanced HCC treated with CyberKnife SBRT in which a subgroup with bile duct invasion was included, but BDTT-specific outcomes were not separately reported. BDI, bile duct invasion; BDTT, bile duct tumor thrombus; BCLC, Barcelona Clinic Liver Cancer; BDR, bile duct resection; DCR, disease control rate; HR, hazard ratio; ICI, immune checkpoint inhibitor; LC, local control; MC, multicenter; MRgRT, magnetic resonance-guided radiotherapy; MST, median survival time; NR, not reported; OS, overall survival; PBT, proton beam therapy; PSM, propensity score matching; PVTT, portal vein tumor thrombosis; RFS, recurrence-free survival; TACE, transarterial chemoembolization.

**Table 2:** Baseline Patient and Tumor Characteristics (Immediately Before MR-guided Radiotherapy).

Characteristic	Patient 1	Patient 2	Patient 3	Patient 4
Age (years)	71	78	67	70
Sex	Male	Male	Male	Male
Underlying liver disease	Alcohol-related cirrhosis	Non-B non-C (past HBV)	HBV + alcohol	HBV
Child-Pugh class (score)	B (8)	A (5)	A (5)	A (6)
Albumin (g/dL)	2.8	3.7	3.9	3.4
Total bilirubin (mg/dL)	4.9	0.8	1.3	1.0
INR	1.29	1.06	1.12	1.12
AFP (ng/mL)	27.9	2345	17.27	3268
PIVKA-II (mAU/mL)	685	5937	152	38
BCLC stage	B	B	B	C
Hepatic tumor (size, cm)	S4 (3.0)	S8 (7.2) + S4 (2.7)	S5/8 (2.5)	S4 + right lobe
BDTT location	Left BD + hilum	S8 IHD	Right BD (S5/8)	Right IHD + left HD
Ueda classification	Type II	Type I	Type IIIa	Type IIIa
Central/Peripheral	Central	Peripheral	Central	Central
PVTT	No	No	No	Yes (right PV)
Pre-RT biliary drainage	Yes (ERBD)	No	No	Yes (ERBD)
Prior TACE (sessions)	7	1	6	9

AFP, alpha-fetoprotein; BCLC, Barcelona Clinic Liver Cancer; BD, bile duct; BDTT, bile duct tumor thrombus; ERBD, endoscopic retrograde biliary drainage; HBV, hepatitis B virus; HD, hepatic duct; IHD, intrahepatic bile duct; INR, international normalized ratio; PIVKA-II, protein induced by vitamin K absence or antagonist-II; PV, portal vein; PVTT, portal vein tumor thrombosis; RT, radiotherapy; S, hepatic segment; TACE, transarterial chemoembolization.

**Table 3:** Treatment Details and Clinical Outcomes.

Variable	Patient 1	Patient 2	Patient 3	Patient 4
Prescribed dose	50 Gy/5 fx	50 Gy/5 fx	50 Gy/5 fx	40 Gy/5 fx
BED_10_ (Gy)	100	100	100	72
GTV (cm^3^)	16.7	26.3	15.4	201.1
Normal liver volume (cm^3^)	1355.8	1558.4	1365.6	988.5
Liver Dmean (Gy)	13.87	14.36	12.97	13.58
Liver V15 (cm^3^)	534.2	607.8	495.0	389.0
Liver V21 (cm^3^)	290.1	395.1	254.0	240.2
RT target	Mass + BDTT	BDTT only	Mass + BDTT	Mass + IHD
Best response (mRECIST)	PR	CR	CR	PR
Jaundice resolution	Yes	N/A	N/A	Yes
AFP: pre-RT → nadir (ng/mL)	27.9 → 17.3	2345 → 8	17.27 → 7.82	3268 → 262
Peak total bilirubin (mg/dL)	17.5 †	5.8 ‡	11.8 §	5.1 †
Max RT-attributed bilirubin toxicity (CTCAE)	Not attributed †	Grade 0	Grade 3 (transient) §	Not attributed †
RILD	None	Focal parenchymal changes on MRI *	None	None
RT-related death	None	None	None	None
Post-RT systemic therapy	Atezo/Bev × 3	None	Atezo/Bev × 3 → Lenv	Atezo/Bev × 3
Overall survival (months)	7.5	29 (censored) ¶	22	5.5
Cause of death	Tumor-related hemobilia in the setting of progressive disease	AWD at cutoff	Lung mets bleeding	HCC progression + sepsis

† Peak bilirubin elevation occurred in the context of confirmed tumor progression at 7 months (Patient 1) and 3 months (Patient 4) after MRgRT and was not attributed to radiation toxicity. ‡ Peak bilirubin elevation in Patient 2 (5.8 mg/dL at 12 months after MRgRT) was attributable to cholangitis from an incidentally identified common bile duct stone. It resolved within 13 days after endoscopic stone removal and was not attributed to radiotherapy. The maximum bilirubin observed prior to this event was 1.2 mg/dL, below the upper limit of normal. § Transient grade 3 elevation (peak 11.8 mg/dL, 8.4 times the upper limit of normal) approximately two weeks after MRgRT, attributed to radiation-induced transient cholestasis after multidisciplinary review including hepatobiliary consultation; resolved spontaneously to near-baseline levels (1.5 mg/dL) over three months without biliary intervention. * Focal radiation-induced hepatic parenchymal changes along the radiation port on MRI at 4 and 5 months after MRgRT, without associated hepatic dysfunction. Resolved by 8 months without intervention. ¶ Alive with disease at data cutoff (31 December 2025); overall survival censored at 29 months. Normal liver volume, Dmean, V15, and V21 were calculated for the normal liver structure (total liver volume minus GTV). For Patient 2, normal liver was defined as total liver minus GTV (BDTT only), consistent with the treatment planning structure. AFP, alpha-fetoprotein; Atezo/Bev, atezolizumab plus bevacizumab; AWD, alive with disease; BDTT, bile duct tumor thrombus; BED_10_, biologically effective dose (α/β = 10); CR, complete response; CTCAE v5.0, Common Terminology Criteria for Adverse Events; Dmean, mean radiation dose; fx, fractions; GTV, gross tumor volume; IHD, intrahepatic bile duct; Lenv, lenvatinib; mRECIST, modified RECIST; N/A, not applicable; PR, partial response; RILD, radiation-induced liver disease; RT, radiotherapy; V15/V21, volume of normal liver receiving ≥ 15/21 Gy.

## 4. Our Experience: MRgRT for HCC with BDTT

### 4.1. Patient Characteristics

Five patients with unresectable HCC and BDTT were identified from the institutional registry between January 2019 and December 2025. One patient was excluded because PVTT, rather than BDTT, was the primary treatment target. The remaining four patients underwent MRgRT between January 2023 and December 2024. All patients were male, with a median age of 70.5 years (range, 67–78). Underlying liver disease included alcohol-related liver disease (*n* = 1), hepatitis B virus (HBV) infection (*n* = 1), HBV combined with alcohol-related liver disease (*n* = 1), and non-B non-C hepatitis with a prior history of HBV infection (*n* = 1). All patients had liver cirrhosis. All patients had an Eastern Cooperative Oncology Group performance status of 0–1. Three patients had Child-Pugh class A liver function (scores 5, 5, and 6), and one had Child-Pugh class B (score 8). The latter patient presented with a serum total bilirubin of 4.9 mg/dL secondary to biliary obstruction and had required endoscopic retrograde biliary drainage (ERBD) before MRgRT. This value represented a point on a steadily declining trajectory from a peak of 13.0 mg/dL that followed ERBD, performed approximately two and a half weeks before the first fraction. By the first fraction of MRgRT, the bilirubin level had decreased to 4.0 mg/dL, and by the final fraction to 3.6 mg/dL. Because no established bilirubin threshold for radiotherapy in BDTT exists, the decision to proceed was based on the trend of bilirubin decline together with multidisciplinary assessment of hepatic functional reserve based on Child-Pugh class and biochemical parameters, rather than on a predefined threshold value. Patient 4 similarly presented with obstructive jaundice and cholangitis secondary to bile duct invasion and underwent ERBD before MRgRT. Following biliary drainage, the serum total bilirubin had normalized to 1.0 mg/dL by the time of treatment. Serum total bilirubin levels at MRgRT initiation ranged from 0.8 to 4.9 mg/dL across the cohort. All patients presented with elevated baseline serum AFP levels, ranging from 17.27 to 3268 ng/mL. Three patients had elevated levels of prothrombin induced by vitamin K absence-II (PIVKA-II) (range, 152 to 5937 mAU/mL), whereas one patient had a baseline PIVKA-II within the normal range (38 mAU/mL). BDTT extent was characterized using both classification systems. According to the Ueda classification, BDTT was type I in one patient, type II in one patient, and type IIIa in two patients. Using the central/peripheral classification [6], three patients had central-type BDTT (involving the common hepatic or first-order bile duct or the hilum), whereas one patient had peripheral-type BDTT confined to a second-order intrahepatic duct. Classification was assigned based on abdominal radiology review of dynamic MRI (axial and coronal sequences).

Three patients were classified as BCLC stage B, whereas Patient 4 was classified as BCLC stage C owing to concurrent right PVTT. All patients had previously undergone TACE (median 6.5 sessions; range, 1–9). Two patients (Patients 3 and 4) had also undergone prior radiofrequency ablation. No patient had received systemic therapy prior to MRgRT. Clinical and treatment characteristics are summarized in Table 2.

### 4.2. Treatment

**Figure 2:**
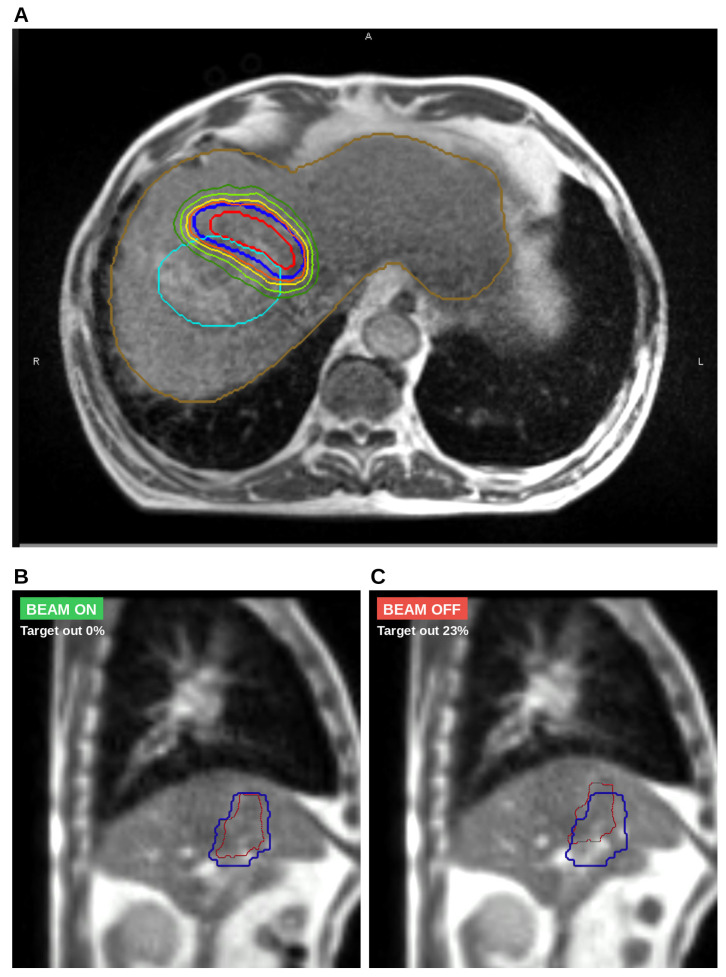
(**A**) Axial MRI acquired on the ViewRay MRIdian 0.35 T MR-linac system at simulation, demonstrating the gross tumor volume (GTV; red) encompassing the intraductal tumor thrombus within the segment 8 intrahepatic bile duct and the planning target volume (PTV; blue) with a 5 mm isotropic margin. The adjacent S8 hepatic mass, delineated separately for documentation purposes only (cyan), was intentionally excluded from the radiotherapy target volume and was addressed by TACE performed approximately three weeks before MRgRT. The normal liver contour is shown in ocher. Isodose lines are displayed as follows: orange, 50 Gy (100%); yellow, 47.5 Gy (95%); yellow green, 40 Gy (80%); green, 25 Gy (50%). (**B**) Sagittal cine-MRI frame acquired during beam delivery with the target within the gating boundary (beam-on). (**C**) Sagittal cine-MRI frame demonstrating target excursion beyond the gating boundary, triggering automatic beam hold (beam-off). In panels (**B**,**C**), the red contour indicates the GTV and the blue contour indicates the PTV boundary. Beam delivery was gated automatically when the target deviated beyond the boundary by more than 3%. GTV, gross tumor volume; PTV, planning target volume; MRgRT, MR-guided radiotherapy; TACE, transarterial chemoembolization.

Target volumes were defined by the treating radiation oncologist in consultation with the multidisciplinary team, taking into account tumor extent, hepatic functional reserve, and response to prior locoregional therapy. In Patients 1, 3, and 4, the parenchymal mass was contiguous with the BDTT, and both components were encompassed within a single target volume. In Patient 4, inclusion of the parenchymal mass was additionally justified because multiple prior TACE sessions had demonstrated progressively reduced tumor vascularity and limited treatment response. In Patient 2, the multidisciplinary team elected to perform TACE targeting the hepatic masses followed by MRgRT directed at the bile duct thrombus. The first TACE session was performed approximately three weeks before MRgRT initiation, and only the BDTT was included in the MRgRT target volume (Figure 2). The prescribed dose was 50 Gy in 5 fractions (BED_10_ 100 Gy) for three patients and 40 Gy in 5 fractions (BED_10_ 72 Gy) for one patient (Patient 4). The dose in Patient 4 was reduced to 40 Gy in 5 fractions as a uniform prescription across the entire PTV, without simultaneous integrated boost. The GTV in this patient (201.1 cm^3^) occupied 20.3% of the normal liver volume (988.5 cm^3^), compared with 1.1% to 1.7% in the other three patients. At 40 Gy, V15 Gy was 389.0 cm^3^, and the spared liver volume fell short of the institutional constraint requiring at least 700 cm^3^ (988.5 − 389.0 = 599.5 cm^3^). Renormalization of the same treatment plan to 50 Gy would have increased V15 Gy to 469.0 cm^3^, reducing the spared liver volume to 519.5 cm^3^ and increasing the normal liver mean dose to 16.98 Gy, exceeding the institutional constraint of 15 Gy. All patients completed the planned treatment without interruption.

### 4.3. Treatment Response and Outcomes

Individual patient courses are detailed in Table 3. With a median follow-up of 14.8 months (range, 5.5–29), OS ranged from 5.5 to 22 months among the three deceased patients. One patient (Patient 2) remained alive at the data cutoff (31 December 2025), with an OS of 29 months from MRgRT initiation. Three patients died: Patient 1 died of tumor-related hemobilia in the setting of progressive disease at 7.5 months; Patient 3 died of hemorrhage from lung metastases at 22 months; and Patient 4 died of HCC progression complicated by sepsis at 5.5 months. No deaths were attributable to treatment-related toxicity.

**Figure 3:**
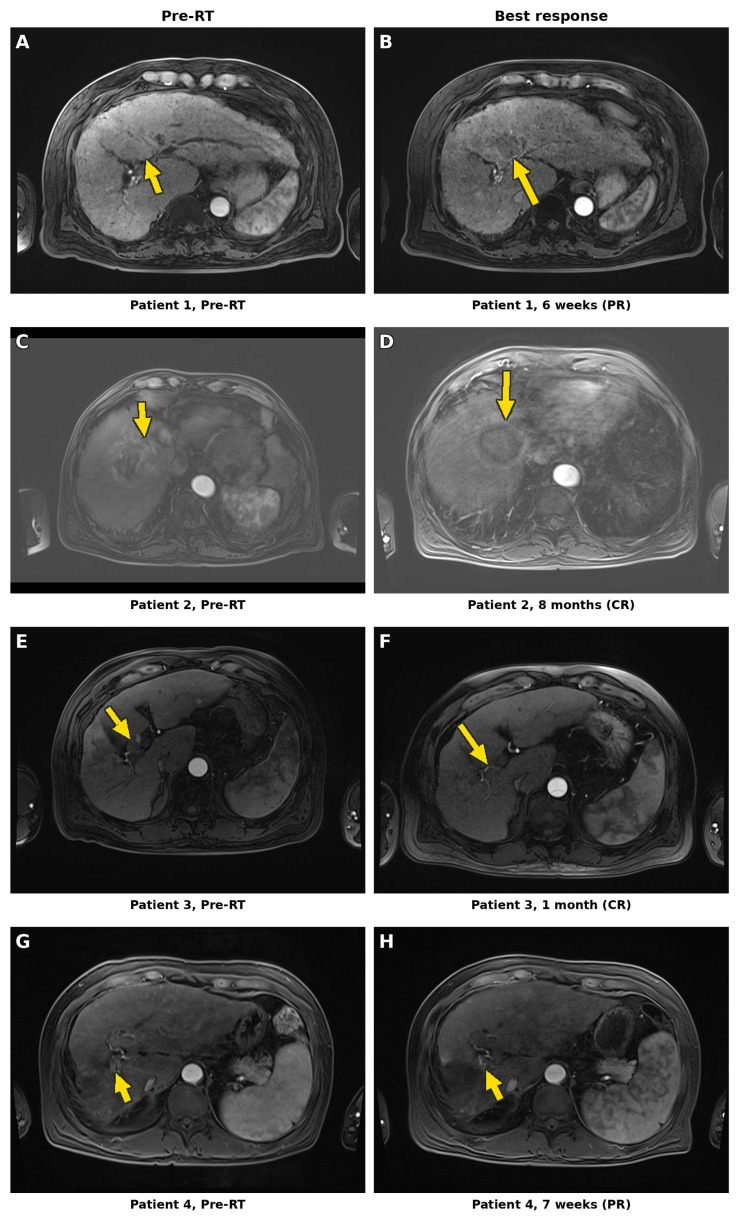
For each patient, the left panel shows contrast-enhanced arterial-phase MRI at baseline (pre-RT) and the right panel shows contrast-enhanced arterial-phase MRI at the time of best response. Yellow arrows indicate the bile duct tumor thrombus. Patients are presented in chronological order of treatment. (**A**,**B**) Patient 1. Pre-RT MRI demonstrating the left bile duct tumor thrombus extending to the hilum with the contiguous S4 mass (3.0 cm) and obstructive jaundice. Post-RT MRI at six weeks showing partial response of the irradiated target with resolution of biliary obstruction. (**C**,**D**) Patient 2. Pre-RT MRI demonstrating the intraductal thrombus within the segment 8 intrahepatic bile duct. The S8 mass (7.2 cm) and S4 mass (2.7 cm) were excluded from the primary target volume. Post-RT imaging at eight months confirming complete response of the irradiated thrombus as well as both non-targeted hepatic masses. Loss of arterial enhancement in all three lesions was already evident on CT performed one week after treatment completion. (**E**,**F**) Patient 3. Pre-RT MRI demonstrating the S5/8 mass (2.5 cm) with right bile duct tumor thrombus. Post-RT MRI at one month showing complete response of the irradiated target with resolution of intrahepatic ductal dilatation. (**G**,**H**) Patient 4. Pre-RT MRI demonstrating the large S4 and right lobe mass with right intrahepatic duct invasion and involvement of the left hepatic duct, and concurrent right portal vein tumor thrombosis. Post-RT MRI at seven weeks showing partial response. A reduced dose of 40 Gy in 5 fractions was prescribed owing to the large target volume and concurrent right portal vein tumor thrombosis. RT, radiotherapy; CR, complete response; PR, partial response.

The best local response was CR in two patients and partial response (PR) in two (Figure 3).

Patient 1 achieved PR on MRI at six weeks, with reduction in the S4 mass from 3.0 to 2.1 cm and improvement in biliary obstruction following irradiation of the left bile duct thrombus and hilar mass. Subsequent imaging assessment was limited by hepatic abscess formation. Extrahepatic progression, manifested by a newly developed portocaval lymph node metastasis, was identified at approximately five months, and Atezo/Bev was initiated as salvage systemic therapy. The patient died of tumor-related hemobilia in the setting of progressive disease at 7.5 months after three cycles of Atezo/Bev.

Patient 2 achieved CR of the targeted intraductal thrombus, confirmed on follow-up MRI at eight months after MRgRT. The adjacent S8 mass (7.2 cm) and S4 mass (2.7 cm), which had been treated with TACE approximately three weeks before MRgRT, also demonstrated loss of arterial enhancement on CT performed one week after MRgRT completion. By three months after treatment, serum AFP and PIVKA-II levels had normalized, decreasing from 2345 to 8 ng/mL and from 5937 to 21 mAU/mL, respectively. The patient remained disease-free for approximately 20 months before developing intrahepatic recurrence within the S8 parenchymal mass, which had been intentionally excluded from the radiotherapy target volume and managed with transarterial chemoembolization. The recurrent lesion received a mean dose of 28.0 Gy (maximum dose 45.7 Gy) within the high-dose planning gradient of the adjacent SBRT field. Following recurrence, additional TACE sessions were administered, and the patient remained alive with disease at the data cutoff (29 months).

Patient 3 demonstrated complete loss of arterial enhancement within the irradiated S5/8 target on MRI performed one month after treatment completion, constituting CR by mRECIST viability criteria. This response was sustained on subsequent follow-up imaging, accompanied by a decline in serum AFP from 17.27 to 7.82 ng/mL. At approximately 12 months, intrahepatic recurrence was detected, comprising a marginal recurrence in segment 8 receiving a mean dose of 33.1 Gy (maximum dose 52.4 Gy), and a separate new lesion in the left lateral section with a mean dose of 7.3 Gy (maximum dose 10.5 Gy), outside the treatment field. The previously irradiated tumor itself remained stable on follow-up imaging, indicating that the intended irradiated target remained durably controlled. The superior lesion, which received near-prescription dose, may represent a marginal recurrence, while the left lateral lesion arose outside the treatment field. Progressive lung metastases subsequently developed. Following recurrence, the patient received Atezo/Bev and subsequently lenvatinib and ultimately died of hemorrhage from lung metastases at 22 months. The spatial relationship between the recurrent lesions and the original treatment volumes is illustrated in Supplementary Figure S1.

Patient 4, who had the largest target volume and concurrent right PVTT, achieved PR at seven weeks after MRgRT. However, rapid intrahepatic progression with complete portal and hepatic vein invasion was identified at three months, and Atezo/Bev was subsequently initiated. The patient died of HCC progression complicated by sepsis at 5.5 months.

Obstructive jaundice resolved in both patients who presented with biliary obstruction.

### 4.4. Toxicity

**Figure 4:**
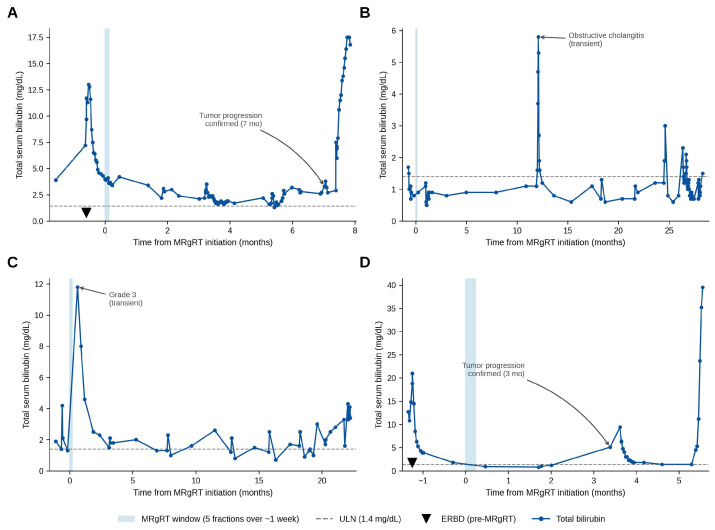
Serial total serum bilirubin concentrations (mg/dL) for each patient (**A**–**D**), plotted from available pre-treatment assessments through last available follow-up. The *x*-axis represents time relative to MR-guided radiotherapy initiation (months); *x*- and *y*-axis scales differ between panels to accommodate the differing follow-up durations and bilirubin ranges. The shaded region indicates the MR-guided radiotherapy treatment window (5 fractions over approximately 1 week). The dashed horizontal line indicates the upper limit of normal (1.4 mg/dL). Triangles indicate the timing of endoscopic retrograde biliary drainage performed prior to MR-guided radiotherapy in Patients 1 (**A**) and 4 (**D**). Bilirubin elevations in Patients 1 and 4 after the treatment window occurred in the context of confirmed tumor progression at 7 and 3 months after MR-guided radiotherapy, respectively, and were not attributed to radiation toxicity. A transient bilirubin elevation in Patient 2 at approximately 12 months after MRgRT (peak 5.8 mg/dL, (**B**)) was attributable to cholangitis from an incidentally identified common bile duct stone. It resolved within 13 days after endoscopic stone removal and was not attributed to radiotherapy. The maximum radiation-attributed bilirubin elevation was grade 3 (Patient 3; (**C**)), a transient elevation that resolved spontaneously without biliary intervention. ERBD, endoscopic retrograde biliary drainage; MRgRT, MR-guided radiotherapy; ULN, upper limit of normal.

No treatment-related deaths occurred. The maximum radiation-attributed bilirubin elevation was grade 3 (Patient 3; peak 11.8 mg/dL, corresponding to 8.4 times the upper limit of normal), representing a transient elevation that peaked approximately two weeks after MRgRT and resolved spontaneously to near-baseline levels (1.5 mg/dL) over the subsequent three months in parallel with radiographic tumor response. After multidisciplinary review, including hepatobiliary consultation, this elevation was attributed to radiation-induced transient cholestasis, although this attribution remains inferential in the absence of biliary intervention. Endoscopic biliary drainage was considered but deemed unnecessary, and the patient was managed entirely in the outpatient setting with oral ursodeoxycholic acid and a course of oral antibiotics. No biliary drainage, stenting, or hospitalization was required. The maximum radiation-attributed bilirubin toxicity was grade 0 in Patients 1, 2, and 4. In Patient 2, the maximum bilirubin prior to the cholangitis event (1.2 mg/dL) remained below the upper limit of normal, and the subsequent elevation to 5.8 mg/dL at 12 months was attributable to cholangitis from a common bile duct stone rather than radiation toxicity, as detailed in Table 3. In Patients 1 and 4, peak bilirubin elevations of 17.5 mg/dL and 5.1 mg/dL, respectively, occurred concurrently with tumor progression at 7 and 3 months after MRgRT and were classified as disease-related rather than radiation-attributed (Figure 4). No patient developed classic or non-classic radiation-induced liver disease. Patient 2 demonstrated focal radiation-induced hepatic parenchymal changes along the radiation port on follow-up MRI at 4 and 5 months after MRgRT, without associated hepatic dysfunction. This finding had resolved by 8 months without intervention. The hepatic abscess observed in Patient 1 was attributed to tumor necrosis with secondary infection rather than radiation toxicity, in keeping with the maintained local response at the irradiated target.

## 5. Discussion

The present series reports MRgRT for unresectable HCC with BDTT. Among four consecutively treated patients, all completed the planned course without interruption. Objective local response was achieved in all patients, with CR in two and PR in two. In addition, obstructive jaundice resolved in all patients presenting with biliary obstruction, and no deaths were attributable to treatment-related toxicity. Collectively, these findings support the feasibility and suggest a favorable preliminary safety profile of MRgRT in patients with HCC and BDTT.

As detailed in the preceding literature review, the management of HCC with BDTT remains poorly standardized. Accumulated evidence indicates that surgical resection is applicable to fewer than 11% of patients [4,6], while TACE and systemic regimens are often limited by hyperbilirubinemia and biliary complications [7,8,9]. Until the present series, locoregional radiotherapy had been evaluated only in two dedicated PBT series comprising 35 patients in total [10,11]. The present series addresses the paucity of evidence regarding effective locoregional options in this population (Table 3).

The technical capabilities of MRgRT are well suited to the central hepatobiliary location of BDTT. MRgRT enables continuous on-board cine-MRI throughout beam delivery, with automatic gating when target motion deviates beyond the planned boundary [12]. This facilitates the use of tighter PTV margins while reducing incidental dose to adjacent biliary mucosa, the portal vein, and the gastroduodenal wall, all of which represent critical dose-limiting organs at risk in central hepatobiliary SBRT [13]. The 5 mm GTV-to-PTV margin in this series reflects the elimination of respiratory motion from the margin calculation through real-time cine-MRI gating, with the expansion accounting solely for residual setup and target delineation uncertainty. No in-field failure was observed in any of the four patients. The adequacy of this margin warrants validation in a larger cohort, and prospective studies incorporating systematic margin analysis are needed. Although direct visualization of the intraductal thrombus on 0.35-T cine-MRI is limited by low-field soft-tissue contrast, MR-based guidance provides clear visualization of the surrounding hepatic parenchyma and adjacent gastroduodenal structures for continuous monitoring during beam delivery. Target delineation can be further refined through fusion with high-resolution diagnostic MRI acquired at simulation [12]. Building on our previous experience with MRgRT for HCC with PVTT in a series of 34 patients [14], the present study extends the application of this approach to the management of BDTT. Real-time cine-MRI gating addresses intrafraction respiratory motion, the dominant source of geometric uncertainty during beam delivery to central hepatobiliary targets. Unlike abdominal targets subject to substantial interfraction organ displacement, the relative positions of intrahepatic structures are generally stable between fractions, and the five-fraction course delivered over consecutive weekdays further minimized the opportunity for clinically meaningful anatomical change. Setup imaging confirmed adequate target coverage at each fraction, and online adaptive replanning was not required.

Biliary toxicity represents the principal safety concern when delivering radiotherapy to the central hepatobiliary tract. As reviewed in Section 3.5, cholangitis was the predominant treatment-related toxicity reported in the PBT series by Lee et al. [10]. In the present series, no treatment-related deaths and no cholangitis-related complications were observed. The maximum radiation-attributed bilirubin elevation was grade 3 in Patient 3 and represented a transient elevation that resolved spontaneously without biliary intervention. No patient required endoscopic or percutaneous drainage for radiation-related toxicity. Although focal radiation-induced hepatic parenchymal changes were observed on MRI in one patient, these imaging abnormalities resolved spontaneously without accompanying biochemical or clinical evidence of radiation-induced liver disease. Bilirubin elevations observed in Patients 1 and 4 were attributable to tumor progression rather than radiation injury, as detailed in Section 4.4. The more favorable toxicity profile observed in the present series, compared with the published proton series, may reflect the combined effects of real-time gating to reduce off-target dose delivery, selective pre-treatment biliary drainage in patients with active cholangitis or hyperbilirubinemia, and differences in patient selection and prescribed dose. Whether more protracted fractionation schedules would reduce the risk of biliary toxicity for centrally located BDTT remains an open question. The bile duct is the treatment target in this setting, and high doses to the biliary mucosa are inherent to therapy regardless of the fractionation schedule employed. The largest published fractionated proton therapy series reported higher rates of cholangitis-related complications [10], although direct comparison across modalities and cohorts is not possible. Therefore, the optimal fractionation strategy that balances durable local control against biliary toxicity in centrally located BDTT warrants further investigation.

Compared with the PBT series, survival outcomes in our cohort were heterogeneous. However, direct comparison is substantially confounded by differences in case mix, as our cohort included one patient with Child-Pugh class B liver function and a pre-treatment bilirubin level of 4.9 mg/dL, as well as one with a large multi-segment target treated at a reduced dose of 40 Gy because of concurrent right portal vein thrombosis. Such patients were underrepresented or excluded in the published proton series. Among the two patients with Child-Pugh class A liver function and no concurrent portal vein thrombosis, Patient 2 survived 29 months (alive at data cutoff) and Patient 3 survived 22 months, outcomes broadly comparable to those in the proton literature. Moreover, MRgRT delivered on a 0.35-T linear accelerator platform is more widely accessible than PBT, which remains confined to specialized centers with substantial infrastructure requirements.

Patient 2 demonstrates an individualized multimodal treatment strategy in which TACE and MRgRT were selectively applied to distinct anatomical targets within the same liver. The multidisciplinary team elected to direct TACE at the hepatic parenchymal masses and MRgRT exclusively at the intraductal thrombus, with TACE performed approximately three weeks before initiation of MRgRT. Early post-treatment CT demonstrated loss of arterial enhancement in both hepatic masses. CR of all treated sites was confirmed on follow-up MRI at eight months. This radiographic response was accompanied by normalization of AFP and PIVKA-II levels by three months. The patient remained disease-free for 20 months before developing intrahepatic recurrence within the S8 mass, which had been treated with transarterial chemoembolization rather than radiotherapy. Given the treatment sequence, the response of the hepatic masses is most reasonably attributed to the preceding TACE, although a contributory effect from incidental radiation exposure within the high-dose falloff gradient of the adjacent SBRT field cannot be excluded. In contrast, because the intraductal thrombus was the sole MRgRT target, its response is more plausibly attributable to radiotherapy. Nevertheless, the relative contribution of each modality cannot be determined from this single case. In Patient 4, by contrast, the parenchymal mass was intentionally included within the radiotherapy target volume because repeated TACE had demonstrated progressively diminished vascular access and limited treatment response. Taken together, these contrasting cases highlight the importance of individualized target volume selection based on prior treatment efficacy and disease distribution. In both published PBT series, the GTV routinely encompassed both the parenchymal mass and the BDTT [10,11]. The complementary TACE-plus-thrombus-only-irradiation strategy employed in Patient 2 has not been previously reported and may offer a liver-sparing alternative when the parenchymal mass responds adequately to transarterial therapy. The intrahepatic recurrence in the non-targeted S8 mass observed at 20 months warrants cautious interpretation.

The recurrence patterns observed in Patients 2 and 3 merit further consideration. Registration of the recurrence imaging with the original treatment plans demonstrated that both recurrences occurred in high-dose regions outside or at the boundary of the delineated gross tumor volume, with mean doses of 28.0 Gy and 33.1 Gy to the recurrent lesions in Patients 2 and 3, respectively. This pattern is consistent with marginal or near-field failure arising from microscopic tumor extension beyond the MRI-visible gross tumor boundary rather than a technical failure attributable to the 5 mm PTV margin. The irradiated targets themselves remained durably controlled in both patients, supporting the adequacy of the MRgRT delivery. Whether PTV margin expansion, dose escalation to at-risk regions, or broader target inclusion would reduce marginal recurrence in this setting warrants prospective evaluation.

The role of ICI-based therapy in this population warrants consideration. Among three patients who received Atezo/Bev after MRgRT, sequential administration was feasible in all cases following normalization of bilirubin levels, either following biliary decompression or radiation-induced tumor regression. Patient 3, who achieved CR of the irradiated target and subsequently received Atezo/Bev followed by lenvatinib, survived 22 months from MRgRT. Patient 2 achieved durable disease control without systemic therapy and remained alive at 29 months. However, the favorable baseline liver function and limited disease burden in this patient preclude any inference regarding the independent contribution of MRgRT. Although definitive conclusions cannot be drawn from this small series, the sequential use of systemic therapy after MRgRT, when clinically feasible, represents a reasonable treatment strategy.

Regarding patient selection, our experience suggests that MRgRT may be best suited for patients with Child-Pugh class A liver function in whom BDTT represents the predominant clinical manifestation and adequate biliary drainage can be established before treatment. Patients 2 and 3 exemplify this profile and achieved the most favorable outcomes in the series. Conversely, patients with Child-Pugh class B liver function, large multi-segment target volumes, or concurrent PVTT represent a higher-risk group with a narrower therapeutic window. Patient 1 exemplified this profile, presenting with Child-Pugh class B8 liver function and a pre-treatment bilirubin of 4.9 mg/dL despite prior biliary drainage, surviving 7.5 months following MRgRT. Unlike in PVTT, where a fixed bilirubin threshold can serve as a reliable indicator of hepatic reserve, obstructive hyperbilirubinemia in BDTT reflects the degree of biliary obstruction and may improve rapidly following drainage. Our institutional approach therefore relies on the pattern of bilirubin recovery after biliary decompression, assessed in the context of multidisciplinary evaluation, rather than on a predefined cutoff. Patient 4 exemplified the latter two risk factors, with a large multi-segment GTV encompassing multiple hepatic segments and concurrent right PVTT that necessitated dose reduction to 40 Gy, and survived 5.5 months following MRgRT. In these patients, MRgRT may still be considered for palliation of biliary obstruction, but realistic treatment goals and expectations regarding prognosis should be discussed with patients and their families before proceeding.

This study has several limitations. First, the series comprises four patients treated retrospectively at a single institution, precluding meaningful statistical inference. Second, target volume definitions were heterogeneous, encompassing mass-inclusive, thrombus-only, and large multi-segment strategies that reflected individualized multidisciplinary decision-making rather than a uniform protocol. Third, both the Ueda and central/peripheral classifications were assigned on the basis of the radiological review without pathological confirmation in all patients. In addition, all patients had received prior transarterial therapy, and most subsequently received systemic therapy, such that the independent contribution of MRgRT to the observed responses and survival cannot be isolated from that of multimodal treatment. Because the bile duct was itself the treatment target, radiation-attributed biliary effects could not be radiographically distinguished from tumor-related biliary changes, and the small number of toxicity events precludes any formal dose–response analysis. Dedicated imaging performed with the primary aim of assessing biliary recanalization was not obtained in any patient. Although ERCP was performed in Patients 1 and 4 for pre-treatment biliary drainage and in Patient 2 for stone removal at 12 months, none of these procedures were designed to systematically evaluate recanalization, and findings should be interpreted accordingly. In Patient 3, who did not have a biliary stent, resolution of intrahepatic ductal dilatation on follow-up MRI at one month was consistent with restoration of biliary patency attributable to tumor response. Future studies should incorporate prospective cholangiographic evaluation to determine the effect of MRgRT on biliary patency in this population. Finally, outcomes were censored at the end of the IRB-approved observation period (31 December 2025), and any subsequent clinical course is not reflected in the reported outcomes.

Despite these limitations, the present series provides preliminary evidence supporting further evaluation of MRgRT in this setting, with an emphasis on refining patient selection and treatment strategies. Given the rarity of BDTT, multicenter collaborative registries may represent a more feasible strategy than prospective trials for validating the role of MRgRT in this population.

## 6. Conclusions

To our knowledge, this is the first reported series of MRgRT for unresectable HCC with BDTT, demonstrating consistent feasibility across four patients with varying hepatic reserve. All patients completed the planned course without interruption, objective local responses were achieved in all four cases, and obstructive jaundice resolved in every patient presenting with biliary obstruction. No sustained radiation-attributed biliary toxicity or treatment-related mortality was observed. A single transient grade 3 bilirubin elevation resolved spontaneously without biliary intervention. Although the small sample size and retrospective design preclude definitive conclusions, these findings provide preliminary evidence supporting MRgRT as a feasible treatment strategy with an acceptable short-term safety profile for this rare condition. In conjunction with the reviewed literature, our experience suggests that MRgRT warrants prospective evaluation as a local treatment option for selected patients with unresectable HCC and BDTT, with patient selection guided by hepatic functional reserve and the extent of biliary involvement.

## Data Availability

The datasets generated during and/or analyzed during the current study are not publicly available due to patient privacy but are available from the corresponding author on reasonable request.

## Supplementary Materials

The following supporting information can be downloaded at: https://www.mdpi.com/article/10.3390/curroncol33070425/s1, Figure S1: Registration of recurrence imaging with the treatment plan for the two patients who developed intrahepatic recurrence.

## Abbreviations

The following abbreviations are used in this manuscript:

AFP	Alpha-fetoprotein
Atezo/Bev	atezolizumab plus bevacizumab
BCLC	Barcelona Clinic Liver Cancer
BDR	Bile duct resection
BDTT	Bile duct tumor thrombus
BED_10_	biologically effective dose (α/β = 10)
CI	confidence interval
CR	Complete response
CT	Computed tomography
CTCAE	Common Terminology Criteria for Adverse Events
ERBD	Endoscopic retrograde biliary drainage
GTV	Gross tumor volume
HAIC	Hepatic arterial infusion chemotherapy
HBV	Hepatitis B virus
HCC	Hepatocellular carcinoma
ICI	Immune checkpoint inhibitor
mRECIST	modified Response Evaluation Criteria in Solid Tumors
MRgRT	Magnetic resonance-guided radiotherapy
MRI	Magnetic resonance imaging
OR	Odds ratio
ORR	Objective response rate
OS	Overall survival
PBT	Proton beam therapy
PIVKA-II	prothrombin induced by vitamin K absence-II
PR	Partial response
PTV	Planning target volume
PVTT	Portal vein tumor thrombosis
RFS	Recurrence-free survival
RILD	radiation-induced liver disease
TACE	Transarterial chemoembolization
SBRT	Stereotactic body radiotherapy
