# Supplementary figures and images for: Magnetic Resonance-Guided Radiotherapy for Unresectable Hepatocellular Carcinoma with Bile Duct Tumor Thrombus: A Case Series and Review of Treatment Options
Source: Curr Oncol. 2026 Jul 16;33(7):425. doi: 10.3390/curroncol33070425 (PMC13407657; doi:10.3390/curroncol33070425)

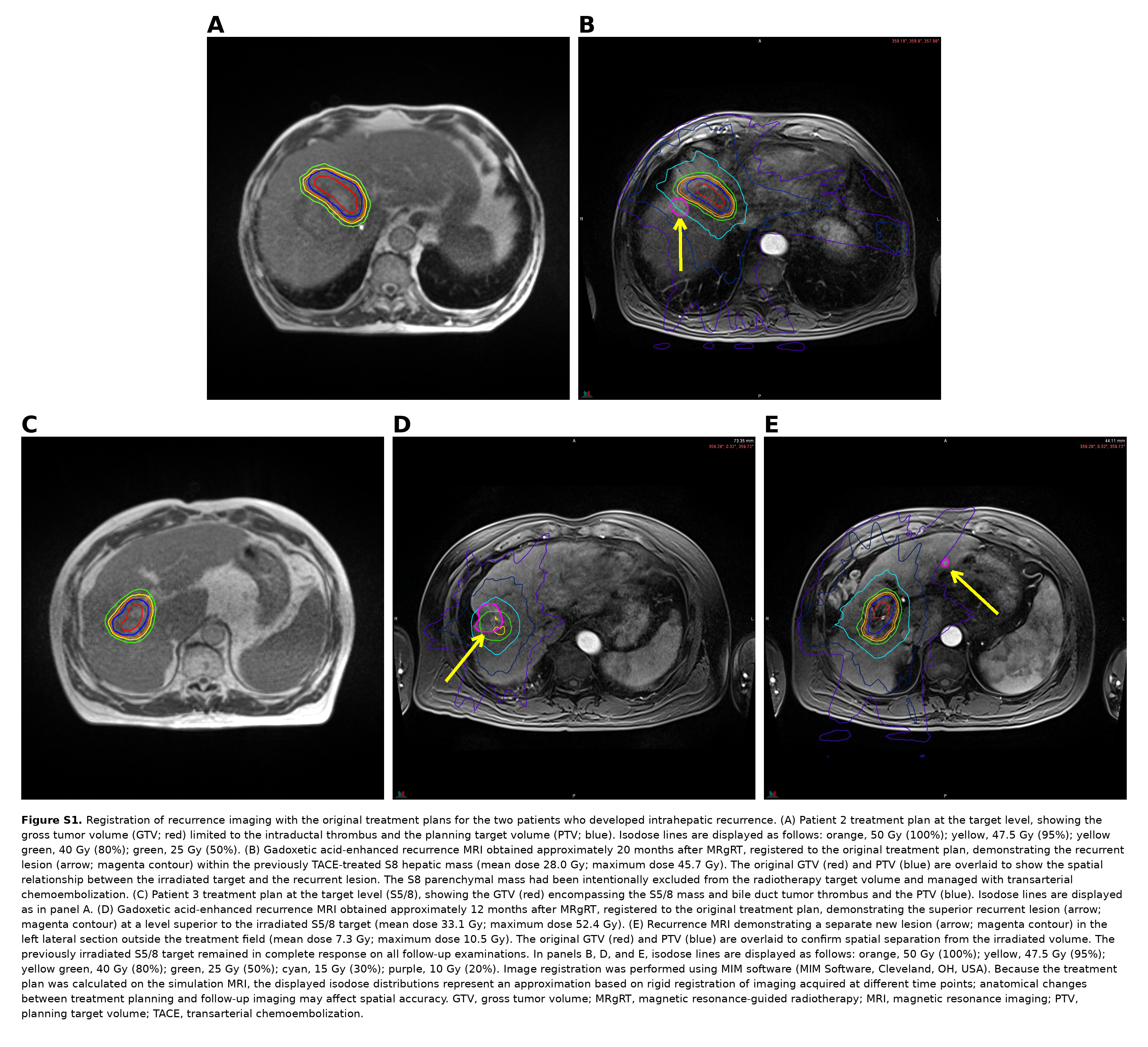

Supplement: Supplementary file 1 [file curroncol-33-00425-s001.zip › Figure_S1_hotdose_revised legend.tiff]
